# Men or Automata? Comments on Paolo Aiello's Painting for TranslationalMedicine@UniSa

**Published:** 2013-01-04

**Authors:** G de Simone

**Affiliations:** Accademia delle Belle Arti di Napoli

## I. Men or Automata?

The painting is a powerful allegory of knowledge and life.

A spiral tower of Babel rises in the back; in the middle, Mercury alias Hermes is standing. Bearer of knowledge (in the alchemic tradition the “Mercury of philosophers” symbolized with his caduceus the synthesis of opposites), messenger of gods and dreams, guide of the souls, he is staring at an egg, a primigenial and universal symbol of the origin of life, which has been pulled out of the body-dummy, lifeless and dissected, by another figure, who is also caught in contemplation. Men or automata? The ambiguity of figures – inspired by noble Renaissance models, such as Mantegna and Dürer – refers to the eternal myth of Pygmalion, the “divine” power to breathe life into inert matter; in other words, the comprehension of the secret functioning of the human mind and body.

## II. Uomini o automi?

L'opera è una potente allegoria della conoscenza e della vita.

Sullo sfondo di una torre a spirale di ascendenza babelica, si erge Hermes alias Mercurio. Portatore di conoscenza (nella tradizione alchemica il Mercurius philosophorum simboleggiava con il suo caducèo la sintesi degli opposti), messaggero degli dei e dei sogni, traghettatore delle anime, osserva assorto l'uovo, simbolo primigenio e universale di origine della vita, estratto dal corpo-manichino ormai esanime e a pezzi da un'altra figura, anch'essa rapita in concentrata contemplazione. Uomini o automi? L'ambiguità delle figure – di nobile ispirazione rinascimentale, tra Mantegna e Dürer – rimanda all'eterno mito pigmalioneo, la facoltà “divina” di infondere la scintilla della vita alla materia inerte; detto altrimenti, la comprensione dei meccanismi più segreti che regolano il funzionamento della mente e del corpo umani.

## Figures and Tables

**Fig. 1.**
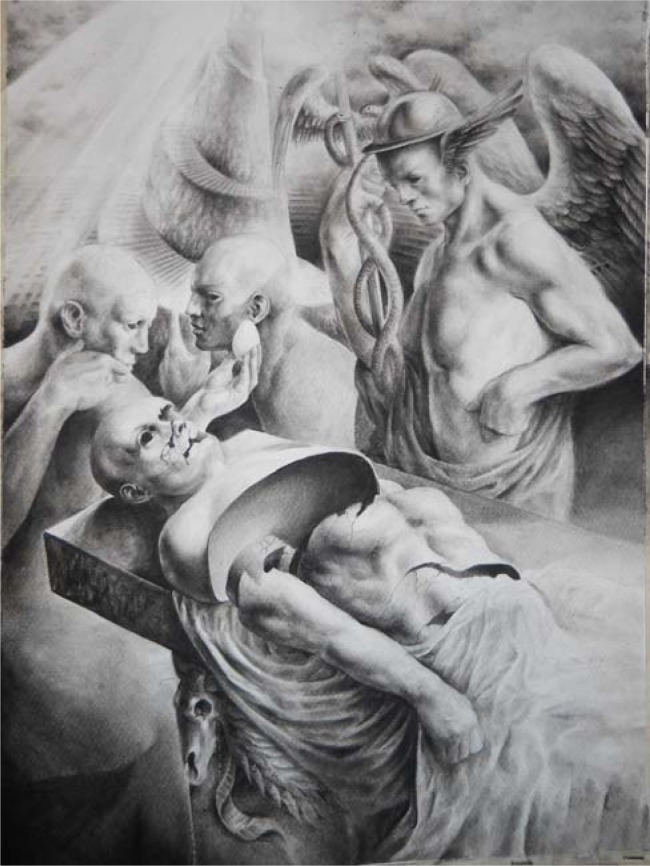
Paolo Aiello. Copertina per la rivista TranslationalMedicine@UniSa. 2012

